# Impact of Preoperative Comprehensive Body Composition on Postoperative Outcomes in Patients with Esophageal Cancer

**DOI:** 10.3390/jcm14207392

**Published:** 2025-10-20

**Authors:** Kiyohiko Shuto, Yoshihiro Nabeya, Mikito Mori, Chihiro Kosugi, Akihiro Usui, Kazuo Narushima, Hiroaki Shimizu

**Affiliations:** 1 Department of Surgery, Teikyo University Chiba Medical Center, Chiba 299-0111, Japan; 2 Division of Esophago-Gastrointestinal Surgery, Chiba Cancer Center, Nitona-cho, Chiba 260-8717, Japan

**Keywords:** esophageal cancer, skeletal muscle, body fat, body composition, prognosis

## Abstract

**Background/Objectives:** Myopenia, myosteatosis, and loss of body fat have been reported as adverse prognostic factors in various malignancies. However, the prognostic value of a composite evaluation of these body composition (BC) parameters remains unclear. The purpose of this study was to investigate the impact of preoperative BC status on postoperative outcomes in patients with esophageal cancer (EC). **Methods**: Seventy patients who underwent curative resection for thoracic EC were retrospectively analyzed. Psoas muscle area, psoas muscle density, and body fat area were measured on preoperative computed tomography. Using sex-adjusted cutoff values, each parameter was assigned a score of 1 if above the cutoff or 0 if below, yielding a composite BC score ranging from 0 to 3. Associations with 5-year overall survival (5y-OS) and postoperative complications were assessed. **Results:** Low muscle mass, reduced muscle density, and low body fat were each associated with poorer survival, with hazard ratios (HRs) of 2.900, 2.909, and 2.990, respectively (*p* = 0.005, 0.028, and 0.002). Patients with unfavorable BC (score ≤1) showed significantly worse 5y-OS (25% vs. 72%, *p* < 0.001) and a higher incidence of postoperative severe complications (42% vs. 18%, *p* = 0.028). On multivariate analysis, BC status was identified as an independent prognostic factor (HR = 3.940, *p* = 0.002), comparable to pathological stage (HR = 6.028, *p* = 0.005). **Conclusions:** A composite BC status incorporating skeletal muscle mass, muscle density, and body fat serves as a valuable predictor of short- and long-term outcomes in postoperative EC patients, providing an integrated measure of patient vulnerability.

## 1. Introduction

Esophageal cancer (EC) remains a malignancy with significant morbidity and mortality worldwide [1,2]. Despite advancements in multimodal treatment, including surgery, neoadjuvant therapy, and perioperative care, long-term prognosis remains suboptimal, as evidenced by national registry data showing only moderate improvements in survival over time [3]. In addition to significant surgical stress, neurological dysphagia, gastric tube volume reduction, delayed gastric emptying, and associated reflux symptoms can easily lead to decreased oral intake. Consequently, over 60% of patients experience a 10% or more weight loss within 6 months after EC surgery, and 20% of patients exhibit more than a 20% weight loss, predisposing them to malnutrition [4].

Weight loss and malnutrition occurring before and after treatment are thought to be associated with alterations in body composition (BC), with changes in skeletal muscle comprising approximately 40% of body mass, and in body fat comprising approximately 20% [5], representing the primary contributors to these alterations. Myopenia, a loss of skeletal muscle mass, myosteatosis, a deterioration in skeletal muscle quality due to intramuscular fat deposition and associated with muscle hypodensity, and loss of adipose tissue have each been reported as adverse prognostic factors in various malignancies [6,7,8,9]. Recent studies in patients with EC have highlighted the prognostic significance of these BC components. It has been reported that decreased skeletal muscle mass before treatment is associated with poor prognosis, that skeletal muscle and body fat are prone to decrease during preoperative chemotherapy, and that changes in BC during preoperative chemotherapy increase the risk of postoperative complications and worsen long-term outcomes [10,11,12,13]. Moreover, recent systematic reviews and meta-analysis demonstrated that sarcopenia is significantly associated with both poorer overall survival and increased postoperative complications in patients with EC, underscoring its prognostic importance across different treatment settings and assessment methods [14]. Although many studies have investigated the relationship between BC and prognosis in patients with EC, most have evaluated skeletal muscle mass, skeletal muscle quality, and body fat mass separately, with few studies assessing these BC factors in an integrated manner [15,16]. Comprehensive preoperative assessment of BC may facilitate more accurate determination of the appropriateness of interventions intended to improve BC during preoperative treatment and postoperative management.

Computed tomography (CT) enables precise, site-specific assessment of BC, distinguishing skeletal muscle, subcutaneous fat, and visceral fat. Unlike bioelectrical impedance or dual-energy X-ray absorptiometry, CT allows evaluation of muscle quality via attenuation values, reflecting intramuscular fat infiltration. Furthermore, pre-existing diagnostic or preoperative CT scans can be repurposed for quantitative analysis without additional radiation exposure.

For these reasons, in this retrospective study, we aimed to investigate the association between preoperative comprehensive BC and postoperative outcomes in patients with EC.

## 2. Materials and Methods

### 2.1. Patients

This retrospective cohort study was approved by the Institutional Review Board of Teikyo University (Teirin 18-171) and was conducted in accordance with the principles of the Declaration of Helsinki (2013). The need for informed consent was waived due to the retrospective design of the study because all data were anonymized and analyzed in aggregate. From January 2007 to December 2024, the medical records of patients with EC who underwent thoracic esophagectomy with lymphadenectomy at the Department of Surgery, Teikyo University Chiba Medical Center, were reviewed. A total of 72 patients underwent curative resection for EC during the study period, and all were consecutively enrolled. The inclusion criteria were the availability of complete clinical data, including preoperative non-contrast CT images and blood test results. After excluding 2 cases with incomplete data, 70 patients were included in the final analysis. Of the total 70 cases, 47 (67%) were diagnosed with squamous cell carcinoma (SCC), while the remaining 23 (33%) were diagnosed with adenocarcinoma.

### 2.2. Treatment and Follow-Up

Initially, patients underwent routine examinations that included physical examination, blood tests, esophagogastroduodenoscopy, contrast-enhanced CT scan, and upper gastrointestinal series, and were diagnosed histologically with esophageal squamous cell carcinoma (SCC) or adenocarcinoma. Treatment and follow-up were planned according to the treatment guidelines in Japan at the time [17,18]. TNM classification was originally determined according to the Japanese classification guidelines used at that time, and pre- and postoperative treatments were planned accordingly [19,20]. For the present study, however, TNM stages were reclassified according to the 8th edition of the Union for International Cancer Control (UICC) TNM classification [21].

Preoperative chemotherapy was administered to eligible patients with clinical stage II or higher disease, in accordance with the evidence available at the time [22,23,24]. Fourteen patients received two courses of fluorouracil (5-FU, 800 mg/m^2^, days 1–5) plus cisplatin (CDDP, 80 mg/m^2^, day 1), 13 patients received three courses of docetaxel (DTX, 70 mg/m^2^, day 1), 5-FU (700 mg/m^2^, days 1–5) and CDDP (70 mg/m^2^, day 1), and 3 patients received two courses of S-1 (80–120 mg/day for 2 weeks) plus oxaliplatin (L-OHP, 100 mg/m^2^, day 1). In patients who did not receive preoperative chemotherapy, surgery was performed within 4 weeks after the CT scan, whereas in patients who received preoperative chemotherapy, treatment response was evaluated by CT 4 weeks after completion of chemotherapy, and surgery was performed within 2 weeks after the CT scan. Right thoraco-abdominal esophagectomy with lymphadenectomy was performed. The surgical approach, either McKeown or Ivor Lewis esophagectomy, was selected based on the location of the primary tumor, histological type, and the suspected sites of nodal metastasis. Posterior mediastinal gastric conduit reconstruction was the first-choice procedure unless the patient had previously undergone gastrectomy. The thoracic approach was either open thoracotomy (*n* = 54) or thoracoscopic surgery (*n* = 16). There were no cases of robot-assisted surgery, including the abdominal procedure. A catheter jejunostomy was additionally performed according to the patient’s condition and the surgeon’s discretion (*n* = 56). Postoperatively, the patient was treated in the intensive care unit for systemic management. The prevalence of preoperative comorbidities was estimated using the updated age-adjusted Charlson comorbidity index (CCI) [25,26], and the severity of postoperative complications was determined using the Clavien–Dindo classification system [27].

During the postoperative follow-up surveillance, blood tests were performed every 3 months, CT scans every 6 months, and gastroduodenoscopy annually for 5 years to monitor for recurrence. Post-discharge daily physical exercise was entrusted to the patient. Depending on the patient’s condition and the attending physician’s judgment, oral nutritional supplements were administered either via a jejunostomy catheter or by oral intake. For patients with pathological stage II or higher, postoperative adjuvant chemotherapy was administered depending on the patient’s general condition and preference: S-1 (*n* = 25), 5-FU plus CDDP (*n* = 17), S-1 plus L-OHP (*n* = 4), or nivolumab alone (*n* = 1) [22,24,28]. Patients with recurrence received chemotherapy or chemoradiotherapy, with first-line chemotherapy regimens including 5-FU plus CDDP (*n* = 12), DTX plus 5-FU and CDDP (*n* = 4), S-1 plus L-OHP (*n* = 1), or nivolumab alone (*n* = 1), when feasible. The median postoperative follow-up was 1104 days (interquartile range [IQR], 454–2829 days). One patient (1.4%) was lost to follow-up during the 5-year postoperative period.

### 2.3. Measurement of BC Components

Preoperative CT scans were obtained within 4 weeks before surgery using a 64-slice multidetector CT system (Lightspeed VCT or Revolution EVO; GE Healthcare Technologies Inc., Chicago, IL, USA). Non-contrast images of the upper to mid-abdomen were acquired during a breath hold with the following settings: tube voltage of 120 kVp, tube current of 200–600 mA, spiral pitch of 1.0–1.5, and slice thickness of 5 mm. Unless contraindicated, a subsequent routine contrast-enhanced cervico-thoraco-abdominal scan was performed using non-ionic iodinated contrast agents.

**Figure 1:**
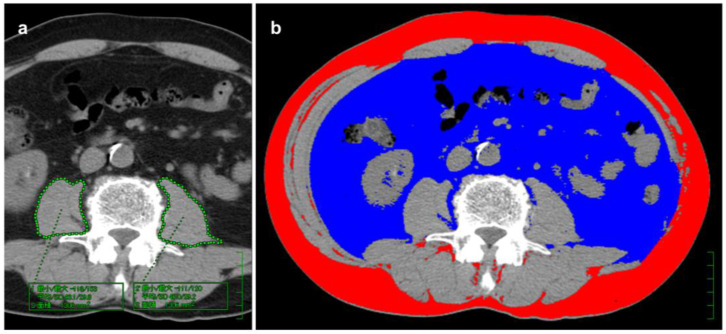
Cross-sectional computed tomography (CT) images at the third lumbar vertebra. (**a**) Psoas major muscles were semi-automatically traced to calculate total muscle area and mean CT density. Green boxes show measured values (maximum/minimum, mean/SD, and region area). (**b**) Body fat volume, including subcutaneous (red) and visceral (blue) fat, was measured using Fat Tissue Analyzer 3.0 (Virtual Place Fujin Raijin, Canon Medical Systems, Tochigi, Japan) with thresholds of −190 to −30 HU.

CT images were transferred to a dedicated workstation for analysis. Two experienced gastrointestinal radiologists (M.M. and K.N.), who were blinded to the clinical results, evaluated cross-sectional non-contrast images at the level of the third lumbar vertebra (L3) to quantify three BC components: skeletal muscle area (cm^2^), muscle attenuation (Hounsfield units [HU]), and total body fat area (cm^2^). The right and left psoas major muscles were semi-automatically traced, and total cross-sectional area and mean CT attenuation were recorded (Figure 1a). Body fat area was calculated as the sum of subcutaneous and visceral adipose tissue using a tissue attenuation range of −190 to −30 HU [29], and analyzed with commercially available software (Fat Tissue Analyzer version 3.0, Virtual Place Fujin Raijin; Aze, Canon Medical Systems, Tochigi, Japan) (Figure 1b). The areas of skeletal muscle and fat tissue were standardized to patient height (cm^2^/m^2^) and used for subsequent evaluation.

### 2.4. Cutoff Values for BC Components and BC Scoring

Sex-adjusted cutoff values for BC parameters were applied for evaluation. The optimal cutoff for each BC component was determined based on survival status using the Youden index derived from receiver operating characteristic (ROC) analysis. Accordingly, the cutoff values for muscle area, muscle density, and fat area were set as follows: males, 5.68 cm^2^/m^2^, 92.9 HU, and 58.6 cm^2^/m^2^, respectively; females, 2.52 cm^2^/m^2^, 80.6 HU, and 20.0 cm^2^/m^2^, respectively. A BC score was then calculated by assigning 1 point for values above the cutoff and 0 points for values below the cutoff.

### 2.5. Statistical Analysis

Continuous variables are presented as medians with IQRs. The significance of differences between two continuous variables was assessed using the Mann–Whitney U test, while comparisons of two categorical variables were performed using the chi-squared test. The correlation was assessed using Spearman’s rank correlation coefficient. Cumulative survival rates were estimated using the Kaplan–Meier method. Prognostic analyses were performed using the Cox proportional hazards model to calculate hazard ratios (HRs) with 95% confidence intervals. A *p*-value of < 0.05 was considered statistically significant. Variables found to be significant in the univariate analysis were included in subsequent multivariate analyses. For prognostic analyses, cutoff values were determined based on survival status using the Youden index derived from ROC analysis, as applied to BC parameters. All tests were conducted using SPSS software (version 28; IBM Corp., Armonk, NY, USA).

## 3. Results

### 3.1. Patient Characteristics

Patient characteristics are summarized in Table 1. Forty-seven patients (67%) were diagnosed with SCC. One patient had clinical stage 0 SCC with multiple T1a lesions. Among the six patients with clinical and pathological stage IV disease, four were classified as stage IVB, presenting with metastases to supraclavicular nodes (No. 104).

### 3.2. Long-Term Outcomes According to BC Status

The measured BC values were as follows: in males, skeletal muscle mass 5.33 [4.33–6.15] cm^2^/m^2^, muscle density 87.1 [77.9–91.2] HU, and fat mass 75.6 [51.6–108.4] cm^2^/m^2^; and in females, skeletal muscle mass 3.67 [2.51–5.53] cm^2^/m^2^, muscle density 73.2 [61.0–81.0] HU, and fat mass 57.1 [30.7–72.8] cm^2^/m^2^. The correlations among BC parameters were as follows: a weak positive correlation between muscle mass and muscle density (R = 0.355, *p* = 0.003), a weak positive correlation between muscle mass and fat mass (R = 0.319, *p* = 0.007), and an almost negligible correlation between muscle density and fat mass (R = −0.193, *p* = 0.110).

**Figure 2:**
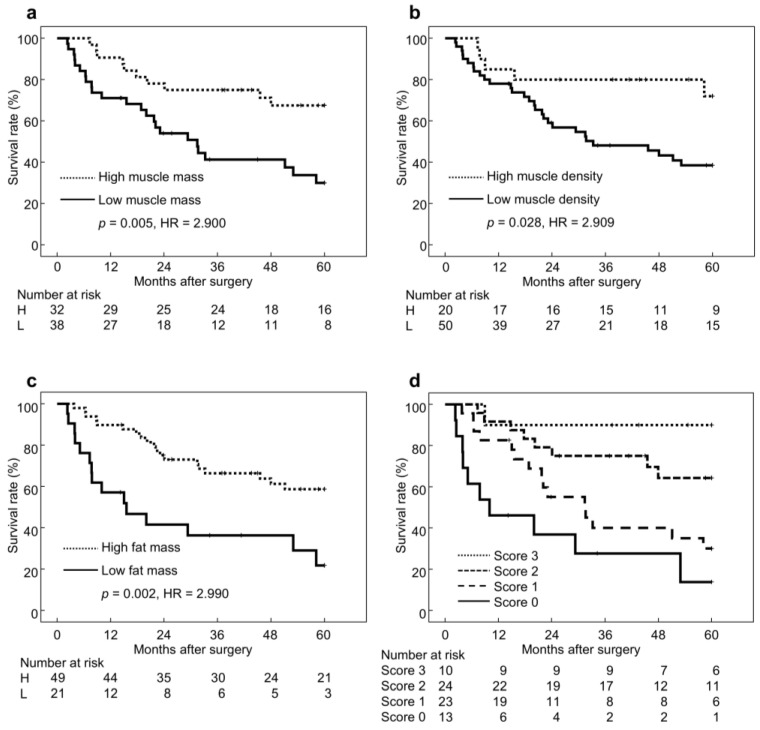
Survival curves stratified by body composition. Abbreviations: H, High; L, Low; BC, Body composition. (**a**) Muscle volume, (**b**) Muscle density, (**c**) Fat volume, (**d**) Body composition score.

Based on the cutoff values of BC parameters, 5-year overall survival (5y-OS) was significantly lower in patients with low muscle mass (30% [*n* = 38] vs. 68% [*n* = 32], *p* = 0.005, HR = 2.900), low muscle density (39% [*n* = 50] vs. 72% [*n* = 20], *p* = 0.028, HR = 2.909), and low fat mass (22% [*n* = 21] vs. 59% [*n* = 49], *p* = 0.002, HR = 2.990), compared with their respective higher groups (Figure 2a–c). According to the BC score, 5y-OS was 14% for score 0 (*n* = 13, HR = 18.294), 30% for score 1 (*n* = 23, HR = 9.378), 64% for score 2 (*n* = 24, HR = 3.750), and 90% for score 3 (*n* = 10, HR = 1). Prognosis improved with increasing BC score (Figure 2d). Because the 5y-OS for all patients was 48%, patients with a BC score ≥ 2 were classified as the favorable BC status group (*n* = 34, 49%), and those with a score ≤ 1 as the unfavorable BC status group (*n* = 36, 51%). The unfavorable BC group showed significantly poorer 5y-OS compared with the favorable group (25% vs. 72%, *p* < 0.001, HR = 4.029).

### 3.3. Patient Characteristics According to BC Status

Table 2 shows the preoperative patient characteristics according to BC status. Although the two groups were comparable in most factors, the unfavorable BC group had a lower body mass index (*p* = 0.039) and a higher CCI (*p* = 0.004).

### 3.4. Long-Term Outcomes in Patients with Recurrence

**Figure 3:**
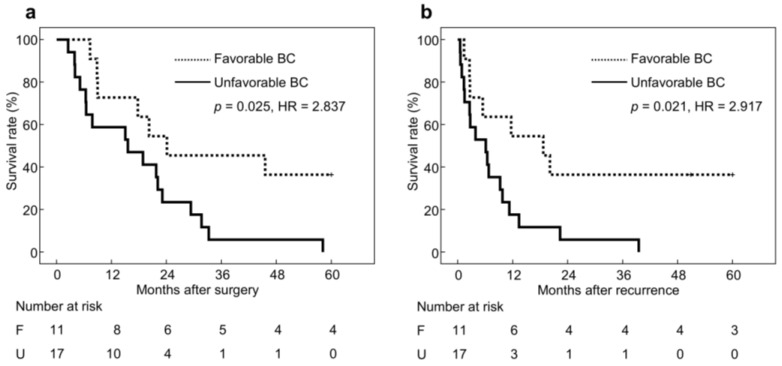
Survival curves stratified by body composition status in patients with recurrence. Abbreviations: BC, Body composition; F, Favorable body composition; U, Unfavorable body composition. (**a**) Overall survival, (**b**) Post-recurrence survival.

Cancer recurrence occurred in 28 patients (11 in the favorable BC group and 17 in the unfavorable BC group). For patients with recurrence, the median postoperative follow-up period was 594 days [IQR, 226–998 days], and the median follow-up period after recurrence was 201 days [IQR, 78–601 days]. The proportion of patients receiving chemotherapy after recurrence was similar in the two groups (*n* = 8, 73% vs. *n* = 10, 59%; *p* = 0.453), and no significant difference was observed in the duration of chemotherapy after recurrence (median [IQR]: 251 [0–1260] vs. 42 [0–226]; p = 0.191). However, in the favorable BC group, five patients continued chemotherapy for more than 1 year and three underwent resection of recurrent lesions, whereas no such cases were observed in the unfavorable group. Overall survival was significantly better in the favorable BC group (36% vs. 0%, *p* = 0.025, HR = 2.837) (Figure 3a), and post-recurrence survival also tended to be better in the favorable BC group (36% vs. 0%, *p* = 0.021, HR = 2.917) (Figure 3b).

### 3.5. Short-Term Outcomes According to BC Status

Postoperative complications by grade were as follows. In the favorable BC group: Grade I or lower, 15; Grade II, 13; Grade III, 5; Grade IV, 1; and Grade V, 0. In the unfavorable BC group: Grade I or lower, 12; Grade II, 9; Grade III, 11; Grade IV, 1; and Grade V, 3. The incidence of Grade II or higher complications was statistically comparable between the groups (56% vs. 67%, *p* = 0.354). In contrast, severe complications of Grade III or higher occurred significantly more frequently in the unfavorable BC group (18% vs. 42%, *p* = 0.028). When focusing on infectious complications such as anastomotic leakage, pneumonia, thoraco-abdominal abscess, subcutaneous abscess, and catheter-related infection, there was no significant difference in the overall incidence of infectious complications between the groups (44 vs. 61%, *p* = 0.155). Similarly, no significant difference was observed in the incidence of complications of Grade II or higher (41% vs. 50, *p* = 0.459), whereas severe complications of Grade III or higher were significantly more frequent in the unfavorable BC group (15vs. 39%, *p* = 0.023) (Table 3).

### 3.6. Prognostic Factor Analysis Including BC Status

Table 4 summarizes the results of prognostic factor analyses based on clinical variables, including BC status. In the univariate analysis, older age (*p* = 0.025), lower serum albumin level (*p* = 0.012), elevated serum C-reactive protein (*p* = 0.043), higher neutrophil-to-lymphocyte ratio (*p* = 0.003), higher CCI (*p* < 0.001), greater intraoperative blood loss (*p* = 0.020), severe postoperative complications (*p* = 0.031), advanced pathological stage (*p* = 0.002), and unfavorable BC status (score < 1) (*p* < 0.001) were identified as significant factors. In the multivariate analysis, advanced pathological stage (*p* = 0.005, HR = 6.028) and unfavorable BC (*p* = 0.002, HR = 3.940) remained independent significant prognostic factors. The thoracic approach, whether open thoracotomy or thoracoscopic, was not identified as a prognostic factor.

## 4. Discussion

In this retrospective cohort study, we investigated the prognostic significance of a comprehensive preoperative BC status in patients undergoing esophagectomy for EC. By integrating three core BC components, skeletal muscle mass, muscle density, and body fat mass, into an additive scoring method, we demonstrated that patients with an unfavorable BC status (score ≤ 1) had significantly poorer overall survival than those with a favorable BC status (score ≥ 2). Moreover, unfavorable BC status was associated with a higher incidence of severe postoperative complications, particularly infectious events, and remained an independent predictor of long-term prognosis in multivariate analysis. These findings highlight the clinical value of an integrative approach to BC assessment, and the stepwise association of the body composition score with long-term outcomes may suggest the potential utility of detailed BC evaluation in individual patients.

Previous research has consistently shown that alterations in BC are linked to adverse outcomes across various malignancies [30,31]. Sarcopenia, defined as the depletion of skeletal muscle quantity and quality, has been widely documented as a poor prognostic factor in gastrointestinal cancers, including EC. Recent systematic reviews have further reinforced the prognostic significance of sarcopenia in EC, highlighting its association with both overall survival and postoperative complications [14]. Previous studies have demonstrated that myopenia is associated with higher postoperative morbidity and mortality, as well as impaired long-term survival [4,6]. Similarly, myosteatosis, reflecting diminished muscle quality due to intramuscular fat infiltration, has been linked to inferior outcomes, independent of muscle mass [7,8]. Several studies have further reported that reduced adipose tissue, both visceral and subcutaneous, is associated with poor tolerance to multimodal therapy and worse prognosis [32,33,34]. However, most prior investigations have assessed these BC components individually, without considering the synergistic impact of their concurrent abnormalities. A limited number of studies have highlighted the potential value of integrated BC assessment, characterized by the presence of myopenia and myosteatosis [15,16], but such approaches remain underexplored. Our study contributes to the growing body of evidence suggesting that an integrated assessment of BC may stratify patients effectively, potentially offering a more comprehensive understanding of host vulnerability.

One noteworthy aspect of this study is that a simple additive scoring method, based on routinely available preoperative CT scans, appears to provide clinically relevant prognostic insights. Because the HRs for 5y-OS were similar for muscle mass, muscle density, and fat mass (2.900, 2.909, and 2.990, respectively), each factor was assigned an equal weight of 1 point. Unlike bioelectrical impedance analysis or dual-energy X-ray absorptiometry, CT imaging is already incorporated into the standard diagnostic and staging work-up in clinical practice. Therefore, this approach may be readily applicable in routine clinical practice, as it does not appear to require additional imaging examinations, radiation exposure, or increased medical costs. Moreover, the integrated BC approach applied in this study is straightforward and feasible for implementation in both high-volume cancer centers and smaller institutions, including those without access to specialized equipment such as bioelectrical impedance analysis or dual-energy X-ray absorptiometry. From a practical standpoint, comprehensive BC assessment enables clinicians to identify patients at high risk for poor outcomes prior to surgery. Such risk stratification is crucial for tailoring perioperative care. For example, patients with unfavorable BC profiles may benefit from targeted interventions, including structured prehabilitation programs, nutritional optimization, and enhanced infection-prevention strategies, particularly in cases where a preoperative window is available, such as during neoadjuvant chemotherapy for advanced disease or in early-stage cases with a sufficient waiting period before surgery. Furthermore, BC status could inform surgical decision-making, such as the choice of surgical approach or consideration of less invasive alternatives. In the context of multimodal therapy, knowledge of a patient’s BC profile may also help anticipate treatment tolerance and inform potential adjustments to chemotherapy and/or radiotherapy regimens, including adjustments to drug selection, dosing, or scheduling, as well as the timing of surgery, where appropriate.

The observed association between an unfavorable BC status and an increased incidence of severe complications, particularly infectious complications, warrants further mechanistic investigation. Patients with reduced skeletal muscle mass are predisposed to respiratory complications due to impaired ventilatory muscle strength [6], whereas diminished muscle quality has been linked to systemic inflammation and immunosuppression [7,8]. Adipose tissue depletion reflects limited energy reserves, further exacerbating vulnerability to catabolic stress in the postoperative setting [9]. Collectively, these changes in BC compromise the host’s ability to withstand surgical trauma and recover effectively. Patients who experience postoperative complications exhibit reduced survival, with postoperative pneumonia and anastomotic leakage in particular being associated with worse outcomes [35,36]. Our findings suggest that patients with unfavorable BC are more likely to develop severe infectious complications, which may in turn contribute to poorer long-term outcomes. Unfavorable BC may also increase the risk of both cancer-specific and non-cancer-related mortality and may be associated with overall patient vulnerability. Indeed, unfavorable BC has been associated with low BMI and high CCI, further underscoring its close relationship with global patient frailty. Poor BC status may serve as a clinical manifestation of systemic inflammation, altered cytokine profiles, and metabolic dysregulation, which have been recognized as key drivers of tumor progression and metastasis [37,38]. Additionally, it has been reported that patients with low BC reserves may have reduced tolerance to adjuvant chemotherapy or salvage therapies after recurrence, thereby compromising long-term disease control [39,40]. In our cohort, patients with unfavorable BC had significantly worse post-recurrence survival. Although poorer chemotherapy continuity after recurrence was presumed to be a contributing factor, no significant difference in the duration of post-recurrence chemotherapy was observed between the favorable and unfavorable BC groups. As the median duration was shorter in the unfavorable BC group, a larger sample size may reveal a clinically meaningful difference.

Interestingly, in multivariate analysis, unfavorable BC and advanced pathological stage emerged as independent predictors of survival, suggesting the complementary contributions of tumor- and host-related factors to patient outcomes. While tumor stage reflects cancer burden, BC reflects the host’s capacity to respond to treatment and withstand physiological stress. These findings indicate that BC represents a distinct and clinically significant dimension of risk, independent of tumor stage. In this study, both esophageal adenocarcinoma and SCC were included, as we focused on patients who underwent thoracic esophagectomy for EC. All patients underwent surgery with curative intent under comparable perioperative management protocols, and thus both histological subtypes were analyzed together. Previous studies have shown that differences in histological subtype may influence tumor biology, treatment response, and prognosis; however, these effects are generally less pronounced after curative resection compared with the impact of tumor stage and patient-related factors such as postoperative swallowing function, nutritional management, and maintenance of quality of life [41,42,43]. Consistent with these reports, histological subtype was not associated with preoperative BC status or postoperative outcomes in our cohort. Accordingly, the inclusion of both histological types in this study may reflect the real-world composition of surgical cohorts and may enhance the generalizability of our findings. Regarding surgical approach, whether open thoracotomy or thoracoscopic, was not identified as a prognostic factor. This may be attributed to the small total number of cases and the unequal distribution between the open and thoracoscopic groups, considering that intraoperative blood loss was identified as a prognostic factor in the univariate analysis.

Our findings have important implications for the design of perioperative management strategies in EC, as mentioned above. First, routine assessment of BC should be integrated into preoperative evaluation, enabling clinicians to identify high-risk patients early in the treatment pathway. Second, BC status may represent a modifiable target for intervention. Recent documents have shown that multimodal prehabilitation, including resistance training, aerobic exercise, and nutritional supplementation, can improve functional capacity and potentially enhance outcomes in frail surgical patients [44,45,46]. De Felice et al. reported that, although the importance of nutritional care in oncology is widely recognized, its practical integration into clinical workflows remains insufficient. They emphasized the need for structured, multidisciplinary collaboration to ensure nutrition assessment and intervention are systematically embedded in oncologic management pathways [47]. These findings support our results, underscoring that comprehensive evaluation of body composition and nutritional status should be incorporated into perioperative care for patients with esophageal cancer. Whether targeted interventions can improve BC status and translate into survival benefits in patients with EC remains an important area for future research. Additionally, unfavorable BC may guide postoperative surveillance strategies. Patients with compromised reserves might benefit from closer monitoring for complications, enhanced infection control management, and individualized rehabilitation support. In the era of precision medicine, integrating BC assessment with molecular and genomic profiling could further strengthen models for personalized care.

Several limitations of this study should be acknowledged. First, the retrospective single-institution design, which included only Japanese patients, together with the lack of standardization in histological type, reconstructed organ, and reconstruction route, limits the general applicability of our findings. The relatively small sample size further reduces the statistical power, particularly for subgroup analyses. Therefore, external validation in larger, multicenter cohorts is warranted. Second, the cutoff values for BC components were derived from ROC analysis based on survival status, as reported in previous studies [48,49,50]. Standardized threshold setting is needed to ensure consistency across studies and to facilitate clinical implementation. Third, although we demonstrated associations between BC and outcomes, causality cannot be inferred, and residual confounding by unmeasured factors cannot be excluded. Fourth, we lacked data to examine the relationship between our BC score and other frailty indices or performance status scales, which might have enabled a more comprehensive assessment of overall patient condition. We believe that accumulating additional cases and harmonizing detailed background factors for reanalysis represent important tasks for future research.

## 5. Conclusions

This study demonstrates that an integrative preoperative BC score, derived from routine CT imaging, predicts both short- and long-term outcomes in patients with EC undergoing esophagectomy. Unfavorable BC status was associated with higher rates of severe postoperative complications and independently predicted poorer overall survival. These findings suggest the clinical value of comprehensive preoperative BC assessment, with potential implications for risk stratification, perioperative management, and personalized care. Prospective validation and interventional studies are warranted to confirm the utility of BC scoring and to explore its role in improving outcomes for patients with EC.

## Figures and Tables

**Table 1:** Patent characteristics.

Characteristics	
Age, years, median [IQR]	70 [63–73]
Sex	
Male/Female	62/8
Histological type	
Squamous cell carcinoma/Adenocarcinoma	47/23
Main tumor location	
Upper thoracic/Middle thoracic/Lower thoracic	2/24/44
Clinical stage	
0/I/II/III/IV	1/15/11/37/6
Neoadjuvant chemotherapy	
Yes/No	30/40
Thoracic approach	
Thoracoscopy/Thoracotomy	54/16
Esophagectomy	
McKeown/Ivor-Lewis	43/27
Lymphadenectomy	
Three-field/Two-field	31/39
Reconstructed organ	
Gastric conduit/Jejunum/Colon	54/12/4
Reconstruction route	
Posterior mediastinal/Retrosternal	65/5
Pathological stage	
0/I/II/III/IV	7/16/14/27/6
Postoperative adjuvant chemotherapy	
Yes/No	47/23
Home nutritional supplementation ≥ 3 months	
Yes/No	24/46

Abbreviations: IQR, Interquartile range; Three-field, cervico-thoraco-abdominal lymphadenectomy; Two-field, thoraco-abdominal lymphadenectomy.

**Table 2:** Patient characteristics according to body composition status.

Characteristics	Favorable BC	Unfavorable BC	*p*-Value
Age, years	69 [62–73]	70 [63–73]	0.097
Sex, male, *n* (%)	28 (82)	34 (94)	0.112
BMI, kg/m^2^	23.0 [21.2–24.1]	22.1 [20.2–23.9]	0.039
Alb, g/dL	4.0 [3.6–4.2]	4.0 [3.6–4.2]	0.273
CRP, mg/dL	0.3 [0.3–0.3]	0.30 [0.30–0.30]	0.330
TC, mg/dL	187 [152–232]	190 [158–226]	0.481
NLR	1.94 [1.26–2.90]	1.96 [1.28–3.18]	0.296
Visceral obesity *, yes, *n* (%)	19 (56)	17 (47)	0.469
Age-adjusted CCI	6 (18)	18 (50)	0.004
Histology, SCC, *n* (%)	23 (68)	24 (67)	0.930
Tumor location, lower, *n* (%)	13 (38)	13 (36)	0.854
Clinical stage, ≥II, *n* (%)	20 (59)	23 (64)	0.663
Neoadjuvant chemotherapy, yes, *n* (%)	13 (38)	17 (47)	0.448

Abbreviations: BMI, body mass index; Alb, albumin; CRP, C-reactive protein; TC, total cholesterol; NLR, neutrophil-to-lymphocyte ratio; CCI, Charlson comorbidity index; SCC, squamous cell carcinoma. Continuous variables were expressed as median [interquartile range]. * Defined as a visceral fat mass of ≥100 cm^2^.

**Table 3:** Postoperative complications according to body composition status.

Postoperative Complication	Favorable BC	Unfavorable BC	*p*-Value
Grade classification			
All grade, *n* (%)	29 (85)	33 (92)	0.402
Grade II ≥, *n* (%)	19 (56)	24 (67)	0.354
Grade III ≥, *n* (%)	6 (18)	15 (42)	0.028
Infectious complication *			
All grade, *n* (%)	15 (44)	22 (61)	0.155
Grade II ≥, *n* (%)	14 (41)	18 (50)	0.459
Grade III ≥, *n* (%)	5 (15)	14 (39)	0.023

Abbreviation: BC, body composition. * Infectious complications included anastomotic leakage, pneumonia, thoraco-abdominal abscess, subcutaneous abscess, and catheter-related infection.

**Table 4:** Prognostic factor analysis.

Factor	Category	Univariate Analysis		Multivariate Analysis	
		HR [95% CI]	*p*-Value	HR [95% CI]	*p*-Value
Age	≥72 years	2.166 [1.103–4.254]	0.025	0.882 [0.357–2.718]	0.785
Sex	Male	1.012 [0.356–2.874]	0.982		
BMI	≥20.5 kg/m^2^	1.497 [0.729–3.073]	0.272		
Alb	<4.1 g/dL	2.514 [1.220–5.179]	0.012	0.952 [0.358–2.535]	0.922
CRP	<0.5 mg/dL	2.110 [1.025–4.344]	0.043	0.681 [0.274–1.689]	0.407
TC	<190 mg/dL	1.754 [0.878–3.505]	0.112		
NLR	≥2.71	2.797 [1.402–5.463]	0.003	1.693 [0.722–3.971]	0.226
Visceral obesity *	Yes	0.807 [0.412–1.583]	0.534		
Age-adjusted CCI	≥score 5	3.222 [1.633–6.356]	<0.001	2.093 [0.791–5.541]	0.137
Histology	SCC	1.092 [0.532–2.240]	0.811		
Tumor location	Lower	1.236 [0.602–2.536]	0.564		
Neoadjuvant chemotherapy	Yes	1.697 [0.865–3.329]	0.124		
Thoracic approach	thoracotomy	1.378 [0.642–2.958]	0.411		
Esophagectomy	McKeown	1.736 [0.810–3.720]	0.156		
Lymphadenectomy	Three-field ^†^	1.411 [0.734–2.828]	0.288		
Reconstructed organ	Gastric conduit	1.193 [0.519–2.740]	0.677		
Reconstruction route	Retrosternal	1.182 [0.361–3.872]	0.782		
Intraoperative blood loss	≥7.31 g/kg	2.224 [1.132–4.368]	0.020	1.658 [0.753–3.648]	0.209
Postoperative complication	≥Grade III	2.144 [1.071–4.292]	0.031	1.825 [0.817–4.076]	0.142
Pathological stage	≥stage II	5.314 [1.869–15.110]	0.002	6.028 [1.717–21.155]	0.005
Postoperative adjuvant chemotherapy	Yes	2.038 [0.887–4.684]	0.093		
Home nutritional supplementation	<3 months	1.874 [0.848–4.141]	0.121		
Body composition status	Unfavorable (≤score 1)	4.029 [1.871–8.672]	<0.001	3.940 [1.620–9.581]	0.002

Abbreviations: HR, hazard ratio; CI, confidence interval; BMI, body mass index; Alb, albumin; CRP, C-reactive protein; TC, total cholesterol; NLR, neutrophil-to-lymphocyte ratio; CCI, Charlson comorbidity index; SCC, squamous cell carcinoma. Cutoff values were determined based on survival status, using maximally selected rank statistics for continuous variables and the Youden index from receiver operating characteristic analysis for categorical variables. * Defined as a visceral fat mass of ≥100 cm^2^. ^†^ Cervico-thoraco-abdominal lymphadenectomy.

## Data Availability

The datasets generated and/or analyzed during the current study are available from the corresponding author on reasonable request.

## Abbreviations

The following abbreviations are used in this manuscript:

BC	Body composition
EC	Esophageal cancer
SCC	Squamous cell carcinoma
OS	Overall survival
UICC	Union for International Cancer Control
5-FU	5-Fluorouracil
CDDP	Cisplatin
L-OHP	Oxaliplatin
DTX	Docetaxel
CT	Computed tomography
HU	Hounsfield unit
ROC	Receiver operating characteristic
IQR	Interquartile range
HR	Hazard ratio
CI	Confidence intervals
BMI	Body mass index
Alb	Albumin
CRP	C-reactive protein
TC	Total cholesterol
NLR	Neutrophil-to-lymphocyte ratio
CCI	Charlson comorbidity index
